# Effectiveness of instructional videos for enhancing healthcare provider competencies for hypertension management – a pre-post study in primary healthcare settings, Tanzania

**DOI:** 10.1186/s12913-022-08064-5

**Published:** 2022-05-31

**Authors:** Anbrasi Edward, Gibson B. Kagaruki, Frank Manase, Lawrence J. Appel, Kunihiro Matsushita

**Affiliations:** 1 Department of International Health, Johns Hopkins Bloomberg School of Public Health, Baltimore, 21205 USA; 2 National Institute for Medical Research, Tukuyu Medical Research Centre, Moshi, Tanzania; 3 Department of Epidemiology & Biostatistics, Institute of Public Health, Moshi, Tanzania; 4 Kilimanjaro Christian Medical University College, Moshi, Tanzania; 5 Afrique One ASPIRE via Centre Suisse de Recherches Scientifiques en Côte d’Ivoire, Moshi, Tanzania; 6 Community Center for Preventive Medicine, Dar es Salaam, Tanzania; 7 Departments of Epidemiology and International Health, Johns Hopkins Bloomberg School of Public Health, Baltimore, USA; 8 Johns Hopkins School of Medicine, Baltimore, USA; 9 Johns Hopkins School of Nursing, Baltimore, USA; 10 Department of Epidemiology, Johns Hopkins Bloomberg School of Public Health, Baltimore, USA

**Keywords:** Hypertension Management, Healthcare Provider Competency, Instructional Video Training, Tanzania

## Abstract

**Background:**

Inadequate adherence to hypertension (HT) clinical standards by healthcare providers is one of the major barriers for HT management. We examined the effectiveness of four short instructional training videos on HT management.

**Methods:**

Eighteen primary health care facilities were randomly selected using systematic sampling from five districts in the Dar es Salaam region, Tanzania. Pre-post provider knowledge assessments were conducted six months after training and provider performance was measured using patient observations on 8-10 consecutive adult patients per facility. A Screening Quality Index (SQI), comprised of ten HT screening standards, was used to measure adherence.

**Results:**

Pre-post knowledge scores improved significantly, for, time between blood pressure (BP) readings (28.1% to 72.7%, p=0.01), BP threshold for patients with complications (21.2% to 97.0%, *p*<0.001), and lifestyle/dietary counseling (from 36.4% to 97.0%, *p*<0.001). SQI was significantly higher following the training for all provider groups; Nurses (3.0±3.5 to 8.4±1.0, *p*<0.001), Assistant Medical Officers and Medical Officers (3.5±4.1 to 7.6±2.4, *p*<0.001), and Assistant Clinical Officers and Clinical Officers (5.4±3.8 to 8.4±2.0, *p*<0.001). After training, significantly higher adherence was evident for key aspects of managing patients with HT: e.g., counseling on medication (62.1% to 92.7%, *p*=0.002), side effects (41.4% to 56.1%, *p*=0.009), reducing caloric intake (69.0 % to 95.1%, p=0.003), reducing cooking salt (65.5% to 97.6%, *p*<0.01), increasing physical activity (55.2% to 92.7% *p*<0.001), stopping/reducing cigarette smoking (24.1% to 63.4%, p=0.001), and reducing alcohol consumption (24.1% to 68.3%, *p*<0.001). SQI was significantly associated with number of years of provider experience (more than 2 years), type of primary healthcare facility (public facility), and exposure to the training intervention.

**Conclusion:**

Training with short instructional videos can improve provider competency and clinical performance for HT management. The strategy has the potential to enhance effective implementation of HT control strategies in primary care clinics in Tanzania and elsewhere.

## Background

The global burden of hypertension (HT), the leading preventable risk factor for premature death worldwide, has been projected to increase to 1.56 billion by 2025 [1]. Despite major large scale efforts to address the disease burden, substantial disparities between high income countries and low and middle income countries (LMICs) are evident in awareness, treatment and control, while at the same time, there is a decrease of 2.6% in age-standardized prevalence of HT in high income countries but a 7.7% increase in LMICs [2] between 2000 to 2010. Compared to high income economies, the risk of dying from elevated blood pressure (BP) is double in LMICs [3].

Healthcare systems in LMICs are confronted with a myriad of system deficiencies, including scarce resources, inadequacies in healthcare personnel, and low population awareness for effective control of HT. The estimated prevalence of HT in Tanzania is 28%, with higher prevalence in urban than rural settings [4, 5]. Capacity investments to establish guidelines and enhance the supply chain systems and workforce competencies have been implemented through the 10-year National Non-Communicable Disease Strategy launched by the Government of Tanzania. However, care delivery is still suboptimal with poor rates of diagnosis and treatment [6–8]. Aside from chronic health workforce deficits with a density of just 0.022 physicians per 1,000 persons, inadequacies in health provider knowledge competencies have been reported - only 59% have a fair knowledge of HT, and fewer report being ‘comfortable’ in managing patients with HT [5, 9–11]. Results from a national survey of 725 facilities, showed that only 42% had access to HT guidelines [6].

The implications for poor quality care resulting from lack of clinician awareness and availability of guidelines has considerable implications for effective management of HT, as inaccurate BP screening could result in errors of -23.6 to +33 mmHg systolic BP (SBP) and -14 to +23 mmHg diastolic BP (DBP) readings [12, 13]. Poor physician adherence to HT guidelines can also lead to poor quality of care [14–16].

The evidence of clinician training on performance has been inconsistent, necessitating empirical studies on the effect of training strategies especially in LMIC contexts [17]. A systematic review on medical training to improve healthcare provider knowledge and patient outcomes illustrated short term improvements in knowledge (ranging from 4 hours to 8 weeks), but there was no evidence linking training to long term improvement in knowledge, clinical practice or patient outcomes [18]. The need for complementary strategies integrated with training has been proposed for achieving high quality health systems [19].

Clinical guidelines have been used to reduce variation of clinician practice behaviors and ensure quality of care; however, numerous factors have been cited for lack of guideline effectiveness, including their development, dissemination, implementation, and clinician behavior [20]. Inaccessibility to clinical guidelines was the most common barrier for non-adherence reported by 65% of clinicians in a study in Sudan, followed by pressure from a high patient load (45.3%) and too much information (32.7%) contained in guidelines. One of the key considerations highlighted by the Lancet series on HT was to promote the development of HT guidelines with simple algorithms for the spectrum of care delivery for screening, management, and follow-up in resource-constrained contexts [21].

A systematic review on the use of mobile technologies for healthcare delivery showed improvements in diagnosis and management outcomes for several health conditions illustrating the potential of complementary technologies for enhancing physician competencies [22]. A recent exploratory study of HT screening and management in Tanzania, showed variability in knowledge and skills competency between doctors and nurses and improvements in clinician knowledge, following instructional video training [23]. However, this study was conducted in one primary care clinic, did not include post assessments on patient screening and management, and showed considerable variability in knowledge between provider cadres [23].

In recognition of the importance of effective screening and management of patients for HT control, and interest shared by the Dar-se-salaam regional directorate, this study was designed to determine the effectiveness of training through brief instructional videos, on healthcare provider knowledge over an extended follow-up period, and adherence to HT screening and management standards. In addition, the study explored variations in knowledge and adherence by different cadres of healthcare providers in ambulatory primary care facilities in Dar-es-Salaam region, Tanzania.

## Methods

### Study design

This study was conducted in five districts of Dar-es-Salaam region: Kigambone, Ilala, Temeke, Ubungo, and Kinondoni in February 2020 (pre-training assessment and instructional video training) and August 2020 (post-training assessment). The post assessment was planned for 3 months after the introduction of the videos but postponed due to the pandemic of COVID-19.

A total of 50 health facilities and dispensaries were selected from the 133 facilities in the 5 districts in Dar-es-Salaam region. This was based on the following criteria - functional, at close proximity to the city center (<2h) and an average of 60+ outpatient visits per day, to ensure at least 10 eligible adult patients. The facilities were stratified based on private and public ownership and 20 facilities were randomly selected. The final sample included 12 health centers and 8 dispensaries. However, only 18 of the 20 randomly selected health facilities were included in the final sample, as providers from one facility did not attend the training, and one facility was closed for renovations after the pre-training assessment. Sample size was estimated based on average outpatient visits and adherence improvements of 10%, assuming a baseline prevalence of 30% and an alpha error of 0.05.

Dispensaries are at the bottom of the health system pyramid and are staffed by a clinical assistant and nurse/s depending on the catchment area. They are expected to provide outpatient care and maternal health services to a catchment area population of 6000-10,000 people. The health center is next in the pyramid, , and serve about 50,000 people, and staffed by clinical officer, doctors and nurses [24]. Health centers provide in-patient and out-patient services and provide diagnostic care and emergency obstetric care.

Assistant clinical officers receive a 2-year certificate in clinical medicine, clinical officers undergo a three-year diploma training and provide both curative and preventive care at the dispensaries, health facilities or district hospitals. Assistant medical officers, undergo a three-year diploma training and receive an additional two-year advanced training. Both Clinical Officers and Assistant Medical Officers can prescribe medications, but Assistant Medical Officers can perform surgeries and c-sections. Medical officers or doctors undergo a five-year degree course with 1 year internship. However, due to staffing shortages, upward or downward vertical task shifting is common and higher level provider cadres are often deployed for lower level tasks, and low level cadres are deployed for higher level responsibilities [25].

### Evaluation of healthcare provider knowledge and performance

We used assessment instruments that were previously standardized for patient observations, healthcare provider knowledge competencies and facility capacity assessments [23]. The quality of HT screening and management was based on the Tanzania National Guidelines for HT. The adult patient exit interview tool validated for the Afghanistan Balanced Score Card National Health Services Performance Assessment was modified to integrate the Tanzania HT standards for patient screening, management, and counseling [26]. The tool was reviewed by an expert team from the Tanzania Regional Health Directorate. Based on Johns Hopkins Institutional Review Board protocols, the patient exit interview was translated into Swahili by a local firm and a certificate of translation was provided to ensure accuracy of content. After the training, the research team field tested the instruments in health facilities in Dar-es-Salaam that were not included in the final study.

Five survey teams, each comprised of two enumerators and one supervisor were trained to conduct the observations and interviews with data entry on tablets. The survey teams comprised of data collectors from the regional and district directorate and CCP Medicine. All the data collectors who performed the patient observations and provider interviews were trained in HT management and were either medical officers, clinical officers, or nurses. They also conducted the patient exit interviews for some health facilities. In other facilities, patient exit interviews were conducted by health technicians (pharmacists, health promoters etc).

Upon arrival at the health facility and obtaining permission from the facility in charge the survey team obtained a list of providers who were scheduled to screen and manage patients for HT. If the facility had more than two providers, the team randomly selected two providers from this list. However, in most facilities, there were only two providers, in which case the team selected both providers.

Provider interviews and patient exit interviews were conducted following the patient consultations. Facility capacity audits were conducted at pre-assessment, with supervisors, to determine capacity and training of healthcare providers, medical commodities (e.g., availability of antihypertensive and other medications), and availability, type, and functionality of BP devices etc. As the capacity for service provision may have differed following covid-19 disruptions, we did not report the baseline data for facility audits.

The following sequence was instituted at post assessment. A consent script was used to enroll eligible patients based on subsequent sampling when they arrived at the outpatient facility. Based on average patient volume of 60 outpatients, including 10-15 new patients for each facility, we selected 8-10 eligible adult patients, aged 30+ years to conduct direct observations and patient exit interviews. Patients who consented to the study were observed at the triage center during HT screening by the nurse if triaging was practiced at the facility. Subsequently, we observed patient screening and HT management and counseling by the health provider (Assistant Clinical Officer/Clinical Officer, Assistant Medical Officer/Medical Officer) in the clinical exam room. All observations in this study were based on patient direct observations. Following the patient visit to the pharmacy or lab, exit interviews were conducted before the patient exited the clinic. Patients who presented for follow up care or emergency patients requiring urgent referral and those with hearing or mental health impairments were excluded from the study. Patients who presented for follow-up visits were not included, as the full HT screening procedures are not routinely performed for follow up patients.

Healthcare provider knowledge was assessed through provider interviews using a structured questionnaire. Direct observations of adherence to patient HT screening standards were conducted using an observation checklist at the Nurse triage and clinical exam room. Likewise, patient management and counseling were also performed using an observational checklist at the clinical exam room. The patient exit interview was conducted using a structured interview outside the clinical exam room before the patient exited the clinic.

Standard procedures were employed for translation and field testing; a certificate of translation was obtained from a credited organization. Ethical clearance was obtained from the National Institute of Medical Research in Tanzania and Johns Hopkins Bloomberg School of Public Health Institutional Review Board. Standard protocols ensured patient and provider confidentiality. Informed consent was obtained from the patients, and signed consent from the healthcare providers and supervisors included in the study. A post-training assessment with new COVID-19 safety guidelines was implemented after securing approval from the two institutional review boards.

*Training of healthcare providers*

The enrolled healthcare providers were invited to attend a half day instructional video training session. The videos, developed by a team at Johns Hopkins University, are comprised of four brief 4-5 minute modules;

1. Why is hypertension important (https://youtu.be/3EMcIVWSmPk)
2. Preparing an individual for blood pressure measurement (https://youtu.be/T9J3RE4Eins)
3. How to diagnose hypertension (https://youtu.be/c8gL5ZGKRxc)
4. What to do after a diagnosis of hypertension (https://youtu.be/0u5O1oehnQk)

Participants also received copies of the videos on an external drive and were instructed to share the content with other health providers in their facilities. Three SMS text reminders were sent to the providers to review the videos during the study period. Pre-post knowledge assessments were administered as part of the provider interviews at baseline (February 2020) and six months after the training session (August 2020). The provider knowledge assessments were conducted in English, and were previously standardized following pre-testing and validation for a similar study conducted in the same context [23].

### Statistical analysis

Descriptive statistics, unpaired (adherence to patient screening) and paired t tests (provider knowledge assessments) were performed to determine differences between before and after the training on quality of care and pre-post provider knowledge competencies. A ten-point screening quality index (SQI) with equal weights was computed for the following quality indicators; patient informed about BP measurement, patient rest of 2-5 min before measurement, patient back supported, patient feet supported, patient feet and legs uncrossed, cuff placed on bare arm, middle of cuff aligned at brachial artery, cuff placed at mid heart level, arm rested on flat surface, and patient quiet, i.e., not speaking, texting, or using cell phone during measurement [23]. Bivariate analysis was performed to determine associations of patient, facility, and provider characteristics with SQI. Variables that were significant were included in the multivariable regression model. A variable was considered statistically significant at p-value <0.05. Analysis was performed using Stata Version 15 software. Models were explored for the collinearity between variables.

## Results

### Provider and patient characteristics

Pre-post knowledge assessments were conducted on 33 healthcare providers who participated in the video training workshop (Table 1). Patient observations were conducted on 166 patients prior to training and 181 patients at the follow-up (Table 2). Compared to baseline where only 18.1% of the patients were screened for BP at triage, 31.5% were screened by nurses at triage at the follow-up assessment.

**Table 1:** Selected characteristics of Healthcare Providers and Health Facilities (Provider Interviews)

**Provider Characteristics**	**N (%)**
Type of Provider	*N*=33
*Assistant Clinical Officer*	1(3.0)
*Clinical Officer*	21(63.6)
*Assistant Medical Officer*	4(12.1)
*Medical Officer*	7(21.2)
Provider Gender	
*Male*	17(51.5)
*Female*	16(48.5)
**Facility Characteristics**	*N*=18
Type of Facility	
*Dispensary*	8(44.4)
*Health facility*	10(55.6)
Facility Management	
*Public*	11(61.1)
*Private*	7(38.9)

**Table 2:** Selected Characteristics of Patients Screened by Healthcare Providers (Patient Direct Observation)

Patient Characteristics	Pre Intervention (*N*=166)	Post Intervention ^a^ (*N*=181)
Gender		
* Male*	61(36.7)	58(32.0)
* Female*	105(63.3)	123(68.0)
Age		
* 30-39 years*	64(38.6)	65(36.5)
* 40-59 Years*	61(36.7)	73(41.0)
* ≥60 years*	41(24.7)	40(22.5)
* Mean (SD)*	47.7(14.4)	47.6(14.3)
Patient BP screening point		
* Pts screened at Triage only*	30(18.1)	57(31.5)***
* Pts screened at Dr.'s room only*	96(57.8)	114(63.0)
* Pts screened at Triage and Dr.'s room*	40(24.1)	10(5.5)

^a^ Different out-patients screened at pre- and post-intervention time periods

***p-value<0.001

### Pre-post healthcare provider knowledge scores

Table 3 illustrates the results from the pre-post provider knowledge assessments. Knowledge competencies improved from pre-assessments, and were statistically significant for the following; reporting adequate knowledge about HT (78.8% to 100%, p=0.005), reporting adequate skills for BP measurement (87.9% to 97.0%, p=0.047), awareness that HT management is lifelong (81.8% to 100%, p=0.010), awareness that HT often shows no symptoms (51.5% to 84.8%, p=0.004), awareness of all standard guidelines for patient preparation prior to BP screening (69.7% to 93.9%,p=0.011 ), correct cuff deflation rate (48.9% to 78.8%, p=0.011), correct time between BP readings (28.1% to 72.7%, *p*<0.01), minimum of 2 BP readings at 1^st^ visit to confirm HT based on guidelines (21.2% to 63.6%, *p*<0.001), BP threshold for patients with diabetes, cardiac or renal impairments (21.2% to 97.0 %, *p*<0.001) and knowledge of all patient counseling standards for appropriate lifestyle and dietary measures to control HT (36.4% to 97.0 %, *p*<0.001).

**Table 3:** Pre-Post Knowledge Differences by Healthcare Provider Cadre (Provider Interviews)

Provider Knowledge Components	Assistant Medical Officer/Medical Officer (***N***=11)		Assistant clinical officer/clinical officer (***N***=22)		Total (***N***=33)	
	Pre (***n***=11)	Post (***n***=11)	Pre (***n***=22)	Post (***n***=22)	Pre (***n***=33)	Post (***n***=33)
***Training***						
Previous in-service training for HT	3(27.3)	11(100)***	7(31.8)	22(100)***	10(30.3)	33(100)***
Type of training received						
* Risks and complications of HT*	2(66.7)	10(90.9)	2(28.6)	20(90.9)**	4(40.0)	30(90.9)**
* Treatment for HT*	1(33.3)	11(100)*	5(71.4)	20(90.9)	6(60.0)	31(93.9)*
* Patient preparation prior to BP measurement*	1(33.3)	11(100)*	2(28.6)	22(100)***	3(30.0)	33(100)***
* Patient monitoring and counselling*	1(33.3)	10(90.9)	2(28.6)	19(86.4)**	3(30.0)	29(87.9)**
**Knowledge awareness**						
* Have adequate knowledge about HT*	9(81.8)	11(100)	17(77.3)	22(100)*	26(78.8)	33(100)**
* Have adequate skills for BP measurement*	10(90.9)	11(100)	19(86.4)	21(95.5)	29(87.9)	32(97.0)*
* Correct cut off for HT Diagnosis (140/90)*	9(81.8)	10(90.9)	14(63.6)	22(100)	23(69.7)	32(97.0)
* BP threshold for patients with diabetes, cardiac or renal impairment should not exceed 130/80*	3(27.3)	11(100)***	4(18.2)	21(95.5)**	7(21.2)	32(97.0)***
* HT management is lifelong*	7(63.6)	11(100)*	20(90.9)	22(100)	27(81.8)	33(100)*
* Consequences of HT (heart attack, stroke, death)*	11(100)	10(90.9)	22(100)	22(100)	33(100)	32(97.0)
* HT Management goal – Reduce SBP/DBP to 140/90*	10(90.9)	9(81.8)	18(81.8)	21(95.5)	28(84.8)	30(90.9)
* Effective treatment can reduce the risk of heart attack, stroke, and death*	11(100)	10(90.9)	21(95.5)	22(100)	32(97.0)	32(97.0)
* Correct definition of HT*	11(100)	11(100)	21(95.5)	21(95.5)	32(97.0)	32(97.0)
* HT often shows no symptoms*	7(63.6)	10(90.9)	10(45.5)	18(81.8)*	17(51.5)	28(84.8)**
* BP seasonal variations; time of day, season, physical activity*	*8(72.7)*	*7(63.6)*	*9(40.9)*	*16(72.7)*	*17(51.5)*	*23(69.7)*
* Heart attack definition*	9(81.8)	10(90.9)	15(69.2)	20(90.9)	24(72.7)	30(90.9)
* BP Screening Standards: back and feet supported, feet uncrossed, cuff placement - midpoint upper arm wrap cuff snugly on bare arm/light clothing -ensure 2 fingers can slip at bottom edge, arm rested on flat surface, rest 2-5m before measuring BP*	8(72.7)	10(90.9)	15(68.2)	21(95.5)*	23(69.7)	31(93.9)*
* Patient sitting quietly during BP measurement does not cause error in reading*	*8(72.7)*	*8(72.7)*	*7(31.8)*	20(90.9)**	*15(45.5)*	28(84.9)*
* Correct recording for BP*	*8(72.7)*	*6(54.6)*	*13(59.1)*	*15(68.2)*	*21(63.6)*	*21(63.6)*
* Correct cuff deflation rate 2mmHG/sec*	*7(63.6)*	*9(81.8)*	*9(40.9)*	*17(77.3)*	*16(48.9)*	26(78.8)*
* Brachial artery for measuring BP*	*8(72.7)*	*7(63.6)*	*15(68.2)*	*17(77.3)*	*23(69.7)*	*24(72.7)*
* Correct cuff application determined by placing 2 fingers under bottom edge of cuff*	*8(72.7)*	*11(100)*	*21(95.5)*	*22(100)*	*29(87.9)*	*33(100)*
* Correct placement of BP cuff – bottom of cuff placed above the bend of the elbow*	6(54.6)	7(63.6)	11(50.0)	14(63.6)	17(53.1)	21(63.6)
* Recommended time between BP readings 1-2min*	*3(27.3)*	*8(72.7)*	*6(27.3)*	16(72.7)*	*9(28.1)*	24(72.7)**
* Correct patient positioning does not include holding arm up in the air*	*8(72.7)*	*7(63.6)*	*14(63.6)*	22(100)**	*22(66.7)*	*29(87.9)*
* Importance of ensuring patient did not smoke, exercise, or drink a caffeinated beverage 30 minutes before BP measurement*	8(72.7)	7(63.6)	18(81.8)	19(86.4)	26(78.8)	26(78.8)
* HT counseling does not include stopping medication if traditional herbs are used*	3(27.3)	4(36.4)	6(27.3)	3(13.6)	9(27.3)	7(21.2)
* Recommended intake of salt/day for patients with HT (5g or 1tsp)*	11(100)	10(90.9)	20(90.9)	21(95.5)	31(96.9)	31(96.9)
Strategies to control HT: *Maintain healthy lifestyle, reduce salt intake, regular BP medication, regular follow up with provider*	11(100)	11(100)	19(86.4)	22(100)	30(90.9)	33(100)
Lifestyle changes to control HT; *Reduce dietary salt <5g/day, daily consumption of fruit/vegetables, reduce tobacco products, lose weight, regular exercise, avoid/limit alcohol*	3(27.3)	10(90.9)**	9(40.9)	22(100)	12(36.4)	32(97.0)***
* The most that healthy diets can reduce SBP is 2mmHg*	*9(81.8)*	*8(72.7)*	*14(63.6)*	*17(77.3)*	*23(69.7)*	*25(75.8)*
**Tanzania Treatment Guidelines for HT**						
* Aware of Tanzania Treatment Guidelines*	8(72.7)	10(90.9)	17(77.3)	20(90.9)	25(75.8)	30(90.9)
* Ministry guidelines for 3 readings of BP - minimum of 2 days apart of 2 months and >140/90*	9(81.8)	9(81.8)	21(91.5)	19(86.4)	30(90.9)	28(84.8)
* Protocol for HT pt follow up (1-3m, every 6m)*	10(90.9)	6(54.6)	17(77.3)	21(95.5)	27(84.4)	27(84.4)
* Minimum of 2 BP readings at 1st visit to confirm HT*	3(27.3)	7(63.6)	4(18.2)	14(63.6)**	7(21.2)	21(63.6)**
* Treatment for HT; lifestyle modification and medicines*	11(100)	11(100)	22(100)	22(100)	33(100)	33(100)
**Aware of first line antihypertensives based on national protocol**						
* Thiazides*	9(81.8)	9(81.8)	9(40.9)	15(68.2)	18(54.6)	24(72.7)
* Beta-Blockers*	3(27.3)	5(45.5)	13(59.1)	13(59.1)	16(48.5)	18(54.6)
* Calcium Channel Blockers*	5(45.5)	6(54.6)	10(45.5)	12(54.6)	15(45.5)	18(54.6)
* ACE Inhibitors*	1(9.1)	5(45.5)	8(36.4)	8(36.4)	9(27.3)	13(39.4)
* Blockers (ARBs)*	1(9.1)	1(9.1)	2(9.1)	2(9.1)	3(9.1)	3(9.1)

*p-value<0.05, **p-value<0.01, ***p-value<0.001

Only 30.3% of the providers reported receiving prior training on HT; of these, 40.0% mentioned training on risks and complications of HT, 60.0% mentioned knowledge of treatment protocols for HT, and only three providers out of 33 had received training on the guidelines for patient preparation prior to BP measurement and patient follow up and counseling.

Results from provider perspectives on the value and functionality of the instructional training videos showed that there was strong agreement/agreement for all elements, exceeding 70% for appropriateness, illustration of objectives and organization and delivery of concepts (Table 4). Two elements showed significant differences between provider cadres; vocabulary used in the narration ([Assistant] Medical Officers 81.8% vs [Assistant] Clinical Officers 100%, *p*<0.05) and pace of narration ([Assistant] Medical Officers 72.7% vs [Assistant] Clinical Officers 100%, *p*<0.05). Providers reported watching all the videos on an average of 4.6 times during the six-months between pre- and post-training assessments, and 87.9% reported sharing the videos/youtube links with other providers within and outside the facility.

**Table 4:** Healthcare Provider Perspectives on Instructional Video Training (Post Intervention Provider Interview)

Perspectives on Instructional Video Training	Assistant Medical Officer/Medical Officer (***N***=11)	Assistant Clinical Officer/Clinical Officer (***N***=22)	Total (***N***=33)
	n(%)	n(%)	n(%)
Reviewed videos since workshop	11(100)	22(100)	33(100)
If Yes, number of times (mean+/-SD)	4.5(1.0)	4.8(0.6)	4.7(2.9)
Shared video link with other providers	8(72.7)	21(95.5)	29(87.9)
Intent to include video content in clinical practice	11(100)	22(100)	33(100)
Strongly Agree/Agree:			
* Accuracy of video content*	10(90.9)	22(100)	32(97.0)
* Appropriate for patient age*	11(100)	22(100)	33(100)
* Appropriate for patient sex*	11(100)	21(95.5)	32(97.0)
* Appropriate for patient ethnicity*	11(100)	21(95.5)	32(97.0)
* Appropriate for physically impaired*	11(100)	21(95.5)	32(97.0)
* Appropriate for local values*	11(100)	21(95.5)	32(97.0)
* Appropriate dress code*	10(90.9)	21(95.5)	31(93.9)
* Appropriate for language*	11(100)	22(100)	33(100)
* Appropriate for social class*	11(100)	22(100)	33(100)
* Introduction motivating stimulated interest*	11(100)	22(100)	33(100)
* Objectives and key elements were clear*	11(100)	22(100)	33(100)
* Simplified complex tasks and avoided introducing unnecessary or irrelevant information*	11(100)	21(95.5)	32(97.0)
* Suggested methods to apply the newly acquired knowledge*	11(100)	22(100)	33(100)
* Allowed to reflect on clinical practice during the viewing*	11(100)	22(100)	33(100)
* Illustrations in video aided learning*	11(100)	22(100)	33(100)
* Learning elements repeated in the conclusion of the video*	11(100)	22(100)	33(100)
* Conducive to learner interaction*	11(100)	22(100)	33(100)
* Well organized and structured*	11(100)	22(100)	33(100)
* The visual quality did not detract from the overall message and content*	11(100)	20(90.9)	31(93.9)
* The vocabulary of the narration was appropriate for the intended audience*	9(81.8)	22(100)*	31(93.9)
* The speed of the narration was slow enough to be understood*	8(72.7)	22(100)*	30(90.9)
* Sound effects added emphasize on certain aspects and enhanced learning*	11(100)	21(95.5)	32(97.0)
* The terms were adequately defined*	11(100)	22(100)	33(100)

*p-value<0.05, **p-value<0.01, ***p-value<0.001

### Adherence to standards of BP measurement from direct patient observation

At triage, where nurses performed the HT screening, adherence to BP screening standards were already high at pre-training and did not improve at post-training for informing the patient that their BP is being measured and waiting 2-5 minutes prior to screening (Table 5). All other screening standards showed significant improvement at triage from the pre- to post-training phase; SQI likewise showed significant improvements from a mean of 3.0 to 8.4 (*p*<0.001) among the nurses.

**Table 5:** Pre-Post Differences in Patient Preparation and Blood Pressure Screening (Patient Direct Observation)

Screening Standards	Pts screened by Triage Nurse		Patients Screened by AMO/MO		Patients screened by ACO/CO	
Patient preparation prior to BP screening (automated/manual BP device)	Pre (*n*=70)	Post (*n*=67)	Pre (*n*=48)	Post (*n*=103)	Pre (*n*=88)	Post (*n*=21)
	n(%)	n(%)	n(%)	n(%)	n(%)	n(%)
*Pt informed BP is being measured*	68(97.1)	62(92.5)	19(39.6)	103(100)**	55(62.5)	20(95.2)**
*Pt rested 2-5m before measurement*	62(88.6)	60(89.6)	20(41.7)	92(89.3)***	61(69.3)	20(95.2)*
*Pt back supported*	42(60.0)	53(79.1)*	9(18.7)	69(67.0)***	31(35.2)	20(95.2)***
*Pt feet supported*	61(87.1)	66(98.5)*	19(39.6)	85(82.8)***	56(63.6)	19(90.5)*
*Pt feet and legs uncrossed*	61(87.1)	66(98.5)*	21(43.7)	85(82.5)***	53(60.2)	20(95.2)**
*Cuff on bare arm*	64(91.4)	67(100)*	20(41.7)	89(86.4)***	58(65.9)	20(95.2)**
*Middle of cuff at brachial artery*	59(84.3)	65(97.0)*	20(41.7)	88(85.4)***	54(61.4)	20(95.2)**
*Cuff at mid heart level*	55(78.6)	59(88.1)	20(61.7)	90(87.4)***	56(63.6)	19(90.5)*
*Pt arm rested on flat surface*	41(58.6)	57(85.1)**	19(39.6)	87(84.5)***	53(60.2)	20(95.2)**
*Pt did not speak, text, use cellphone*	40(57.1)	59(88.1)***	21(43.7)	84(81.5)***	54(61.4)	18(85.7)*
**Screening Quality Index mean(+/-SD)**	3±3.5	8.4± 1.0***	3.5± 4.1	7.6±2.4***	5.4± 3.8	8.4± 2.0***
**BP Measurement (Automatic device**)	***N*=60**	***N*=67**	***N*=12**	***N*=53**	***N*=37**	***N*=19**
*30 second rest period between measurements (if >1 reading)*	5(8.3)	45(67.2)***	1(8.3)	53(100)***	21(56.8)	19(100)**
*Document confirmatory measurement (average of 3 measurements)*	3(5.0)	36(53.7)***	1(8.3)	41(77.4)***	22(59.5)	14(73.7)***
**BP Measurement (Manual device**)	***N*=10**	***N*=0**	***N*=36**	***N*=50**	***N*=51**	***N*=2**
*Tightly closed BP cuff valve stem*	9(90.0)	N/A	10(27.8)	48(96.0)***	26(51.0)	1(50.0)
*Correct placement of steth earpieces*	3(300)	N/A	10(27.8)	48(96.0)***	25(49.0)	1(50.0)
*30 second rest period between measurements (if >1 reading)*	5(50.0)	N/A	1(2.8)	27(54.0)***	0(0.0)_	1(50.0)
*Documented confirmatory measurement (average of 3 measurements)*	N/A	N/A	2(5.6)	42(84.0)	0(0.0)	1(50.0)
**Record of BP Readings, Height, Weight**	***N*=70**	***N*=67**	***N*=48**	***N*=103**	***N*=88**	***N*=21**
*Medical record designated place for BP reading*	20(28.6)	34(51.5)**	22(45.8)	47(45.6)	36(40.9)	10(47.6)
*Pts with 1 SBP/DBP reading*	70(100)	67(100)	20(41.7)	103(100)***	60(69.2)	20(95.2)*
*Pts with 2 SBP/DBP reading*	0(0.0)	20(29.9)***	2(4.2)	99(96.1)***	17(19.3)	20(95.2)***
*Pts with 3 SBP/DBP readings*	0(0.0)	1(1.5)	0(0.0)	85(82.5)***	2(2.3)	20(95.2)***
*Pt weight measured*	50(71.4)	45(67.2)	0(0.0)	53(51.5)***	7(7.9)	9(42.9)***
*Pt height measured*	10(14.3)	13(19.4)	0(0.0)	32(31.1)***	4(4.5)	9(42.9)***

*p-value<0.05, **p-value<0.01, ***p-value<0.001^a^ Different out-patients recruited at post intervention phase. Ϯ Screening Quality Indicators. AMO Assistant Medical Officer MO Medical Officer ACO Assistant Clinical Officer CO Clinical Officer

Adherence to clinical standards for patient preparation prior to BP measurements based on clinical observations also showed significant improvements for all standards for both the clinical officer and medical officer cadres (Table 5). SQI improved significantly from a mean of 3.5 to 7.6 (*p*<0.001) for the medical officer cadres, and a mean of 5.4 to 8.4 (*p*<0.001) for the clinical officer cadres at post training.

For patients who were screened with an automated device, significant improvements were evident at the post-training phase, for adherence to standards of ensuring a 30 second rest between BP measurements, and recording BP, as an average of the readings for all provider cadres, including triage nurses. Proportion of patients with two BP measurements (if the first reading was above the cut off for HT), also increased significantly from baseline for all provider cadres, i.e. triage nurse (0% to 29.9%, *p*<0.001), (Assistant) Medical Officers (4.2% to 96.1%, *p*<0.001), (Assistant) Clinical Officers (19.3% to 95.2%, *p*<0.001).

Patient weight and height is routinely measured for computing the body mass index. Though the improvements were significantly higher at post-training for all provider cadres, adherence levels were still sub-optimal (weight: [Assistant] Medical Officers: 0% to 51.5%, *p*<0.001, [Assistant] Clinical Officers: 7.9% to 42.9%, *p*<0.001, Height: ([Assistant] Medical Officers: 0% to 31.1%, *p*<0.001, [Assistant] Clinical Officers: 4.5% to 42.9%, *p*<0.001).

### Adherence to hypertension management standards

Management of patients with elevated BP, SBP ≥140, and/or DBP ≥90, showed significant pre-post improvements in the following: requesting laboratory tests (68.7% to 79.8%, p=0.019), and type of tests ordered - lipid profile (6.5% to 40.0%, *p*<0.001), sodium (0 to 46.7%, *p*<0.001), blood sugar (30.4% to 49.3%, p=0.041), and electrocardiogram (13.0 % to 29.3%, p=0.039) (Table 6). Quality of patient counseling also increased significantly: medication counseling for explaining treatment regimen (from 62.1% to 92.7%, p=0.002), discussion of possible side effects (41.4% to 56.1%, p=0.009), importance of taking medication (79.3% to 95.1%, p=0.041), dietary counseling for reduced salt in cooking (65.5% to 97.6%, *p*<0.01), reduced table salt consumption (58.6% to 97.6%, *p*<0.001), reduced consumption of sodium rich foods (79.3% to 95.1%, p=0.041), reduced caloric intake (69.0% to 95.1%, p=0.003), and lifestyle counseling to increase physical activity or exercise (55.2% to 92.7%, *p*<0.001), cease or reduce smoking (24.1% to 63.4%, p=0.001), and cease or reduce alcohol consumption (24.1% to 68.3%, *p*<0.001). A significant improvement was also evident for coordinating follow up appointments for patients (79.3% to 95.1%, p=0.041) and referrals to a hospital or health facility (13.8% to 48.8%, *p*<0.001).

**Table 6:** Pre-Post Differences in Patient Management for Elevated BP (SBP≥140 and/or DBP ≥90) (Patient Direct Observation)

Patient Management	Patients with elevated BP	
	Pre (*N*=67)	Post ^a^ (*N*=94)
**Laboratory Testing**	**n(%)**	**n(%)**
Pt was prescribed lab tests	46(68.7)	75(79.8)*
Pt received information about the lab tests	37(80.4)	50(66.7)
*Type of test ordered*	*N*=46	*N*=75
*Lipid profile*	3(6.5)	30(40.0)***
*Renal function test*	2(4.4)	4(5.3)
*Sodium*	0(0.0)	35(46.7)***
*Blood Sugar*	14(30.4)	37(49.3)*
*ECG*	6(13.0)	22(29.3)*
*Other (Malaria, Xray, MRDT, Hb, Cervical screening, Urine analysis, FBR, ESR, HIV Test)*	31(67.4)	42(56.0)
Pt was prescribed antihypertensive medications	29(43.3)	41(43.6)
*Type of medicines prescribed*		
*Hydrochlothiazide*	1(3.5)	3(7.3)
*Bendroflumethiazide*	5(17.2)	3(7.3)
*Furosemide*	10(34.5)	12(29.3)
*Atenolol*	2(6.9)	11(26.8)
*Nifedipine*	15(51.7)	27(65.9)
*Amlodipine*	1(3.5)	8(19.5)
*Repace-H (combination of “Losartan potassium 50mg and hydrochlorothiazide 12.5mg”*	2(6.9)	2(4.9)
**Patient Counseling**		
Patient received counseling:	*N*=29	*N*=41
***Medication Counseling***		
*medication regimen (dose and timing)*	18(62.1)	38(92.7)**
*potential side effects of medication*	12(41.4)	23(56.1)**
*importance of taking the medication*	23(79.3)	39(95.1)*
*where to obtain the medication*	28(96.6)	41(100)
***Dietary Counseling***	*N*=29	*N*=41
*Reduce salt in cooking*	19(65.5)	40(97.6)**
*Reduce table salt consumption*	17(58.6)	40(97.6)***
*Reduce consumption of foods rich in sodium*	23(79.3)	39(95.1)*
*Reduce caloric intake*	20(69.0)	39(95.1)**
***Lifestyle Counseling***		
*Increase physical activity or exercise*	16(55.2)	38(92.7)***
*Cease or reduce smoking*	7(24.1)	26(63.4)**
*Cease or reduce alcohol consumption*	7(24.1)	28(68.3)***
**Referral and follow up appointments**		
*Received assistance on follow up appointments*	23(79.3)	39(95.1)*
*Referred to the next level (hospital/health center)*	4(13.8)	20(48.8)***

*p-value<0.05, **p-value<0.01, ***p-value<0.001^a^ Different out-patients recruited at post intervention phase

### Determinants of Patient Screening Quality

Regression analysis showed positive significant associations of SQI with the following patient level factors - secondary education or higher, single/widowed/divorced patients, providers with more than 2 years of experience, and exposure to training (Table 7). An interaction term for provider work experience and facility type, was included, and was significant (*p*<0.001). Specifically, SQI was significantly lower for privately owned facilities compared to publicly owned facilities and lower in the higher wealth quintiles compared to the lowest wealth quintile. In a multivariable model, providers in public facilities and provider experience of less than 2 years, emerged as significant predictors of the improvement in HT screening quality.

**Table 7:** Univariate and multivariate linear regression analysis of factors associated with hypertension screening quality index at pre and post intervention phase

Patient Characteristics	Mean SQI (SD)	Unadjusted Coefficient β(95%CI)	***P***-value	Adjusted Coefficient β (95%CI)	***P***- value
Patient Sex (ref, Male)	6.2(3.3)				
Female	6.8(3.2)	0.54(-0.18-1.3)	0.141	-0.12(-0.8-0.56)	0.713
Marital status (ref, Married)	5.6(3.7)				
Single/divorced/widowed	7.2(2.8)	1.7(1.0-2.4)	<0.001	0.004(-0.8-0.8)	0.993
Patient Education (ref, none/primary)	5.5(3.7)				
Secondary or more	6.9(3.1)	1.2(0.42-2.0)	0.002	-0.07(-0.9-0.8)	0.875
Patient age (ref, <50y)	6.3(3.5)				
50+y	7.0(2.9)	0.71(-0.01 -1.4)	0.054	0.43(-0.23-1.10	0.200
Main presenting symptom (ref, other)	6.3(3.4)				
Weakness, dizziness, headache, chest pain, chest tightness, elevated BP	7.0(3.1)	0.70(-0.004 -1.4)	0.051	0.5(-0.20-0.80)	0.167
Wealth Quintile (ref, lowest/low)	7.1(3.0)				
Middle	6.4(3.4)	-0.66(-1.6-0.29)	0.174	-0.40(-1.2-0.52)	0.423
High	6.0(3.5)	-1.13(-2.0 to -0.24)	0.013	-0.78(-1.6-0.06)	0.070
Highest	6.4(3.2)	-0.70(-1.73-0.32)	0.179	-0.25(-1.2-0.69)	0.603
**Provider & facility characteristics**					
Facility Ownership (ref, public)	7.1(2.9)				
Private	5.7(3.7)	-1.3(-2.0 to -0.62)	<0.001	-3.2(-4.7 to -1.7)	<0.001
Facility level (ref, dispensary)	6.7(3.1)				
Health center	6.5(3.4)	-0.22(-1.0-0.51)	0.544		
Provider cadre (ref, nurse, assistant medical officer, clinical officer, assistant clinical officer)	6.4(3.3)				
Medical officer (doctor)	6.7(3.3)	0.28(-042-1.0)	0.429		
Provider Gender (ref, female)	6.5(3.4)				
Male	6.6(3.1)	0.06(-0.63-0.75)	0.865		
Providers’ work experience (ref, ≤2years)	5.3(3.9)				
>2 years	6.8(3.1)	1.6(0.7-2.5)	<0.001	-0.7(-2.0-0.58)	0.279
Provider work experience and facility ownership	N/A	2.4(0.6-4.3)	0.009	2.8(1.2-4.5)	0.001
Study Phase (ref, Pre intervention)	5.1(3.8)				
Post Intervention	7.9(1.9)	2.8(2.2 -3.5)	<0.001	2.8(1.2-4.5)	<0.001

Wealth Quintile Asset Index: Electricity, as source of lighting, safe water source, cooking fuel: gas, electricity, kerosene, safe sanitation, motor cycle, bicycle, car, refrigerator, sewing machine, television, stove/gas cooker, radio, iron, cell phone, adequate flooring

Screening Quality Index Scores (SQI) 1.Patient informed that BP is being measured 2.Ensure patient rests 2-5m before measurement 3.Ensure patient back is supported 4.Ensure patient feet is supported 5.Ensure patient feet and legs are uncrossed 6.Place cuff on bare arm 7.Align middle of cuff at brachial artery 8.Place cuff at mid heart level 9.Ensure arm is rested 10. Pt does not speak, text, use cellphone during BP measurement

### Patient Exit Interviews

Results from patient exit interviews (Table 8) showed significant improvements in the following: BP measured at visit (90.2% to 100%, *p*<0.001), provider explaining BP reading (10.4% to 66.9%, *p*<0.001), risk factors associated with high BP (35.4% to 55.8%, *p*<0.001), and cut off for HT (16.4% to 30.0%, *p*<0.001). Likewise, improvement in counseling was evident for patients with high BP levels (SBP ≥140 and/or DBP ≥90) - taking medications regularly (52.2% to 86.2%, *p*<0.001), avoiding adding salt to food before eating (61.2% to 79.8%, p=0.011), cease or decrease smoking (29.9% to 50.0 %, p=0.009), and cease or reduced alcohol consumption (31.3% to 54.3%, *p*<0.001). Compared to baseline, a higher proportion of patients reported receiving health messages on HT, and consequences of high BP at the final assessment (25.6% to 38.7%, p=0.010).

**Table 8:** Pre-Post Differences of Patient Feedback of Clinical Visit related to HT (Patient Exit Interview)

***Patient Report:***	Pre Intervention Phase (***N***=164)	Post Intervention Phase ^a^ (***N***=181)	***p*** value
	n(%)	n(%)	n(%)
**BP measured during the visit**	148(90.2)	181 (100)	<0.001
*Health Provider informed results of BP reading*	131(88.5)	166 (91.7)	0.158
*Health Provider informed patient they he/she has BP*	56(34.8)	75 (45.2)	0.055
**Previous history of receiving BP medications**	47(28.7)	57(31.5)	0.481
*taking medication regularly*	23(48.9)	24(42.1)	0.541
**Reasons for not taking medication regularly**			
*Forgot to take them*	3(12.5)	6(18.2)	0.520
*Could not afford to purchase*	6(25.0)	10(30.3)	0.595
*No time to obtain refills*	1(4.2)	0(0.0)	0.129
*Felt better*	11(45.8)	14(42.4)	0.798
*Didn’t think it was necessary*	4(16.7)	2(6.1)	0.218
*Side effects*	2(8.3)	2(6.1)	0.775
**Health Provider provided information on HT**			
*No information*	88(53.7)	43 (23.8)	<0.001
*Explained about BP reading*	17(10.4)	121 (66.9)	<0.001
*Risk factors associated with BP*	58(35.4)	101(55.8)	<0.001
*Other specify (important of follow up, BP results, avoiding stress, avoid fats foods and table salts etc)*	16(9.8)	11 (6.1)	0.297
**Aware of cut off for high BP**			
*120/80*	8(4.9)	24 (13.3)	0.004
*140/90*	19(11.6)	32 (17.7)	
**Health provider gave advice if patient had high BP:**	***N*=67**	***N*=94**	
*Follow up regularly with doctor*	51(76.1)	69(73.4)	0.697
*Taking medicines regularly*	35(52.2)	81(86.2)	<0.001
*Avoid adding salt before eating*	41(61.2)	75(79.8)	0.010
*Reduce salt during cooking*	43(64.2)	63(67.0)	0.681
*Reduce foods high in sodium*	44(65.7)	64(68.1)	0.748
*Increase physical activity/exercise*	34(50.8)	60(63.8)	0.097
*Stop or decrease smoking*	20(29.9)	47(50.0)	0.011
*Stop or decrease alcohol*	21(31.3)	51(54.3)	0.004
**Report alternative practices for preventing/treating HT**			
*Not using any prevention measures*	24(51.1)	55(94.8)	<0.001
*Increase exercise/physical activity*	9(19.1)	1 (1.7)	0.002
*Stop or decrease alcohol*	1(2.1)	0 (0.0)	0.264
*Herbal medicines*	8(17.0)	0 (0.0)	0.001
*Advice from Traditional Healer*	0(0.0)	0 (0.0)	-
*Homeopathy*	2(4.3)	0 (0.0)	0.113
*Prayer*	6(12.8)	0 (0.0)	0.006
*Reduce table salt*	11(23.4)	1 (1.7)	0.001
*Reduce salt in food during preparation*	13(27.7)	1 (1.7)	<0.001
*Stop or decrease smoking*	2(4.3)	0 (0.0)	0.127
*Decrease processed food (pickles, preserves, canned foods)*	7(14.9)	2 (3.5)	0.025
Heard health messages on HT and consequences	42(25.6)	70 (38.7)	0.010
Received all medicines during visit	110(67.1)	98 (54.1)	<0.001
Aware of dosage for all medicines	120(85.1)	122 (85.9)	0.942
Aware of timing for all medication	111(79.3)	120 (84.5)	0.417
Aware of duration for all medication	113(81.3)	123 (86.6)	0.362
Informed about side effects	23(16.4)	39 (27.7)	0.032
**Payments made for medicines**			
Cash	56(38.9)	69(52.3)	0.004
Health insurance	39(27.1)	41(31.1)	
No payment	55(34.8)	22(16.7)	
Average payment (Tanzania shillings)	12,363	11,500	0.3535

*p-value<0.05, **p-value<0.01, ***p-value<0.001^a^ Different patients examined at pre and post intervention

## Discussion

This study documented that brief video training modules have the potential to improve healthcare provider competencies for effective screening and management of patients for HT. Specifically, adherence to HT clinical standards for patient preparation prior to screening and measurement of BP improved significantly for all groups of providers who manage HT patients in Tanzania. Patient counseling on medication dosage, adherence, lifestyle, and dietary modifications to control HT also showed significant improvements. Type of facility management emerged as a key predictor of screening quality, as patients who were managed by providers from government owned facilities showed higher adherence to HT screening standards than those from privately owned facilities.

Ensuring healthcare provider competencies is one of the essential prerequisites in HT control programs. Lack of knowledge on HT, and awareness of clinical guidelines was also reported by healthcare providers in Tanzania in earlier studies [5, 10, 11]. Though our study showed higher levels of awareness of HT, only 30.3% of the providers reported receiving prior training on HT. Considering the current health provider capacity and resource limitations in Tanzania, competency training, using self-paced videos which provide the technical standards and process of care, are a highly feasible, scalable, and contextually relevant strategy. This can partially address the urgent public health need to expand HT training in Tanzania. However, the content needs to be streamlined to the current guidelines of the national HT control strategies.

A study on HT adherence by doctors in Sudan also illustrated that lack of access to clinical guidelines was the most frequently quoted barrier to effective HT management [27]. Another study on primary healthcare physicians in Saudi Arabia, reported that inappropriate distribution of the guidelines, lack of training, negative attitudes of physicians toward guidelines, and high patient volume were deterrents to guideline use in clinical practice [28]. The same study showed that clinical training on HT and having a lower patient load were significantly associated with higher adherence to standard guidelines.

Competency training and access to clinical guidelines are two of the more popular strategies for enhancing clinician competencies [29]. High cost of training investments, and reproduction and distribution of clinical guidelines to all healthcare providers is an immense strain for health systems in LMICs which face chronic health workforce deficits and financial constraints [30]. Training duration, the methods and medium used, complexity of the information provided are essential considerations as illustrated in a recent systematic review on healthcare provider performance [17].

A cross-sectional study of 150 doctors in Sudan showed that longer duration of clinical experience of healthcare providers was the sole significant positive predictor of high adherence to clinical guideline in mutlivariable regression analysis, a finding which is consistent with our observation. The importance of training and reinforcement of guidelines was recommended to improve clinical governance [27]. Length of physician experience and type of patient (presence of comorbidities and other complications) has also been shown to influence adherence to guidelines [31]. We also observed that providers from government facilities showed significantly higher adherence to clinical standards than those in private facilities, illustrating the need for further investigation on the incentive and healthcare environments, as other studies have shown better performance of the private sector in some aspects [32, 33].

Factors contributing to low clinician adherence to standard clinical guidelines have not been adequately explored. A study on clinician feedback at point of care showed that guideline recommendations were not followed as the recorded BP was not representative of the patient’s typical BP, HT was not the clinical priority for the visit, or patients were non adherent to medications [34]. Another study conducted in the United States, concluded that clinicians overestimate their adherence to HT guidelines recommending point-of-service feedback [35]. Automated decision support systems, like the Assessment and Treatment of Hypertension: Evidence Based Automated Decision Support System (ATHENA-HTN), have been recommended to address the barriers related to knowledge, attitude and behavior barriers to clinician non-adherence [34].

Healthcare systems in LMICs that have minimal access to advanced automated technologies need to rely on inexpensive mechanisms like wall posters, clinician job aids and YouTube video sources that can be readily accessed through smart phones. Guideline concordance may be improved if clinician training is complemented with additional educational aids. For example, the application of mobile technologies in healthcare delivery has shown improvements in physician diagnosis and patient outcomes for several health conditions [22]. Clinicians using guidelines and an mHealth app in Lebanon showed higher adherence for screening patient’s medical history, enquiries for medication complications, lifestyle counseling, scheduling follow up visits and recording of body mass index [36].

Poor adherence to antihypertensive medication is a significant risk factor for cardiovascular disease, and thus patient counseling on medication adherence is crucial in HT management. A survey on US adults reported that only one in four patients received advice on specific lifestyle and behavior changes [37]. In a recent qualitative study in Tanzania, both healthcare providers and patients with HT mentioned poor counseling as a barrier for medication adherence [38]. Our study showed significant improvements (52.2 to 86.2%, *p*<0.001) in patient medication counseling following the video training. Likewise, lifestyle counseling (increased physical activity, reduced smoking, and alcohol, reduced caloric intake, reduced use of cooking salt, and avoiding adding salt to food before eating) all improved at the end of the study.

With the rapid increase of NCD burden in Africa [39], Tanzania like other countries in the region, has developed a more aggressive strategy for addressing systemic issues in the Non-Communicable Disease Action Plan for 2020-2025, with four strategic outcomes: 1. Strengthened leadership, governance, multi-sectoral collaboration and accountability for prevention and control of NCDs 2. Strengthened national capacity for NCD prevention and health promotion targeting all modifiable risk factors (Alcohol, Tobacco, Nutrition, Physical Inactivity, environmental risk factors such as air pollution) and other social determinants, 3. Health systems re-oriented to address NCDs through promotive, preventive, curative, and rehabilitative services 4. Strengthened national capacity for NCD surveillance and research for evidence-based planning monitoring and evaluation. Though Tanzania has made enormous strides to address NCDs instituting school-based interventions, and HT awareness campaigns, concerns persist. Specifically, there is low provider knowledge for NCD management and suboptimal capacity - only half of health facilities that should be able to provide HT care are able to do so. Furthermore, there is a need for integrated strategies to address HT, diabetes and HIV, as well as the requisite training to achieve quality care for these conditions [40].

Our study illustrates the potential for use of accessible and inexpensive technology to provide in-service training to health providers in the primary healthcare setting to enhance their knowledge and skills competency for HT management. The improvements in triage nurse BP screening, though not empirically evaluated, and dissemination of the videos to providers who did not participate in the training offers further evidence that the videos were valued.

### Limitations

Lack of a control group and the clinical behavior change due to observations (namely providers may behave differently due to observations) pose inherent study limitations. Interrupted time series design with a randomized comparison group and follow up assessments to determine if improvements are sustained would provide additional rigor and insight. Future goals for enhancing knowledge competency must include all provider groups engaged in the care continuum, including the triage nurses and the non-communicable disease coordinators. Though we obtained information on supervision of services and other facility capacity factors, e.g. availability of essential medicines and BP measurement devices, these were not considered in the multivariable analysis, posing other limitations.

## Conclusion

Despite national efforts to address the growing burden of HT and bolster system capacity including the establishment of clinical guidelines, care quality remains suboptimal for HT screening and management. The present study provides preliminary evidence that a series of short instructional videos might enhance provider knowledge competencies and adherence to HT screening and management standards. Contextually appropriate solutions can address some of the provider level knowledge and performance barriers that impede care quality but are not a panacea for resolving the broader system constraints that impede effective HT control in LMICs.

## Data Availability

The datasets are available from the corresponding author on reasonable request.

## Abbreviations

BP: Blood Pressure
HT: Hypertension
SQI: Screening Quality Index
